# Physiological and Cellular Ultrastructural Responses of *Isatis indigotica* Fort. under Salt Stress

**DOI:** 10.3390/plants13121593

**Published:** 2024-06-07

**Authors:** Shuang Wu, Xiuwen Jia, Beijing Tian, Feng Zhang, Jingying Zhao, Xinjing Xie, Chenggang Shan, Huimei Wang, Xiaorui Guo, Jinlong Han

**Affiliations:** 1 Key Laboratory of Forest Plant Ecology, Ministry of Education, Heilongjiang Provincial Key Laboratory of Ecological Utilization of Forestry-Based Active Substances, College of Chemistry, Chemistry Engineering and Resource Utilization, Northeast Forestry University, Haerbin 150040, China; 17085347226@163.com; 2 Shandong Academy of Agricultural Sciences, Jinan 250100, China; 3 Shandong Xieshi Chinese Herbal Pieces Co., Ltd., Heze 274000, China; 4 State Key Laboratory of Subtropical Silviculture, College of Forestry and Biotechnology, Hangzhou 311300, China

**Keywords:** salt stress, *Isatis indigotica* Fort., physiological and growth characteristics, ultrastructure

## Abstract

This study aimed to analyze the effects of salt stress on the growth physiology and plant-cell ultrastructure of *Isatis indigotica* Fort. (*I. indigotica*) to evaluate its adaptability under salt stress. The effects of different concentrations of salt (NaCl; 0, 25, and 300 mmol·L^−1^) on the agronomic traits, activities of related enzymes, ion balance, and mesophyll-cell ultrastructure of *I. indigotica* were studied in a controlled pot experiment. Results showed that compared with those of the control group, the aerial-part fresh weight, underground fresh weight, tiller number, root length, root diameter, plant height, and leaf area of salt-stressed *I. indigotica* increased at 25 mmol·L^−1^ and then decreased at 300 mmol·L^−1^. The changes in levels of superoxide dismutase, peroxidase, ascorbate peroxidase, and catalase showed a similar trend, with significant differences compared with control group. Salt stress altered the ion balance of *I. indigotica*, resulting in a significant increase in Na^+^ content and a significant decrease in K^+^ content. The contents of Ca^2+^ and Mg^2+^ changed to varying degrees. The analysis of the microstructure of the root showed that under salt treatment, the epidermal cells of the root significantly thickened and the diameter of the xylem decreased. The results of ultrastructural analysis of mesophylls showed that salt stress can cause cell-membrane contraction, cell-gap enlargement, disorder in the structures of chloroplasts and mitochondria, and an increase in the number of osmiophilic particles. These changes were aggravated by the increase in NaCl concentration. This study reveals the response of *I. indigotica* to salt stress and provides a basis for further study on the salt-tolerance mechanism of *I. indigotica*.

## 1. Introduction

Salt stress is one of the most important issues in global agriculture, especially in arid and semi-arid regions [1]. The presence of excessive salt in soil exceeds the threshold that plants can tolerate, bringing great challenges to the growth and development of plants [2]. Salt stress not only limits the yield and quality of plants but also weakens the ability of plants to further develop and utilize arable land, thereby posing a threat to global food security and the sustainable development of the ecological environment [3]. Under salt stress, plants face multiple challenges, including ion-balance disruption, water stress, oxidative stress, and cell-ultrastructure damage [4]. Excessive salt can enter plant roots, resulting in an imbalance of Na^+^ and K^+^ levels in the cells. This phenomenon disrupts important ion balances and nutrient uptake, interferes with membrane stability and ion-transport processes, and directly affects the normal physiological activities of plants [5]. Previous studies have shown that cotton has successfully adapted to the saline–alkali stress environment through the regulation of the antioxidant enzyme system, the regulation of osmotic substances, and the reconstruction of ion balance [6]. The *AtSIZ1* gene in *Meloidogyne paranaensis* improves the salt tolerance of plants by maintaining ion homeostasis and osmotic balance [7]. A high salt concentration in soil limits the water-absorption capacity of plant roots, resulting in the rapid loss of water from cells. This phenomenon can lead to cell dehydration and atrophy, thus interfering with the plant’s water-regulation ability [8]. For example, some varieties of *Pennisetum glaucum* L. have a slight difference in shoot length, root length, and biomass under salt stress compared with control plants not under salt stress [9]. Under salt stress, the epidermis and endodermis cells of soybean root were thickened, and the number of salt-soluble stomata was increased to reduce water loss [10].

In a certain range of salinity, plants enhance their antioxidant capacity to resist damage caused by oxidative stress [11]. Plants produce more superoxide dismutase (SOD), catalase (CAT), and antioxidant small molecules such as glutathione, vitamin C, and carotenoids, to neutralize active oxidizing substances [12]. Studies have shown that under saline–alkali stress, the activity of SOD in cotton leaves gradually increased, whereas those of peroxidase (POD) and CAT first increased and then decreased [13]. The activities of SOD, POD, and CAT in the roots of *Gossypium hirsutum* L. reached their highest values under NaCl stress [14]. In addition, salt stress has a remarkable effect on the ultrastructure of plant cells, cause responses such as abnormal morphology of chloroplasts, swelling of mitochondria, and thickening of cell walls [15,16]. Salt stress caused swelling of thylakoids in the mesophyll of *S. carnosa* [17].

*Isatis indigotica* Fort. is an herbaceous plant belonging to the Cruciferae family; its leaf (IIL) and root (IIR) are commonly used in traditional Chinese medicines, with anti-inflammatory, analgesic, and antioxidant effects [18,19,20]. Previous studies have focused on the growth, photosynthetic parameters, and antioxidant enzyme responses of *I. indigotica* under salt stress [21], and changes in transcriptomics [22]. However, there are few studies on the growth morphology, mesophyll-cell ultrastructure and root-section changes of *Isatis indigotica* Fort.

This study aimed to explore the growth morphology (fresh weight of aerial part, fresh weight of underground part, tiller number, root length, root diameter, and plant height), activities of antioxidant enzyme (POD, SOD, APX, and CAT), ion content (Na^+^, K^+^, Ca^2+^, and Mg^2+^), ultrastructure of mesophyll cells, and changes in transverse and longitudinal sections of *I. indigotica* roots under different salt concentrations (0, 25, and 300 mmol·L^−1^) to further elucidate the effect of salt stress on *I. indigotica*. These analyses could provide a theoretical basis for improving the salt tolerance of plants.

## 2. Results

### 2.1. Effects of Salt Stress on the Growth of Aerial Part

**Figure 1:**
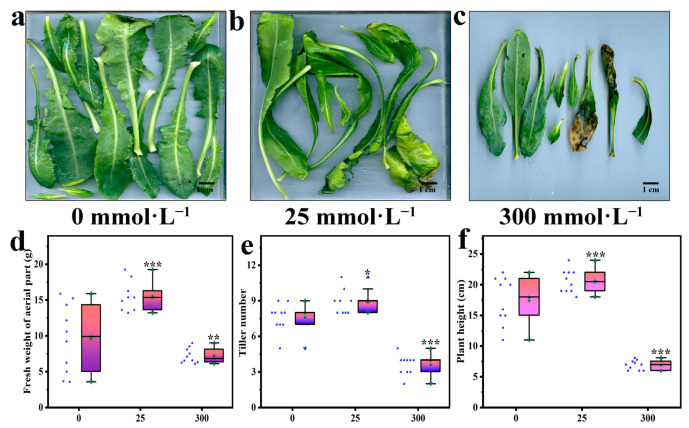
Aerial part of *I. indigotica* under stress from different concentrations of salt. (**a**) Leaf morphology of *I. indigotica* at 0 mmol·L^−1^, (**b**) Leaf morphology of *I. indigotica* at 25 mmol·L^−1^, (**c**) Leaf morphology of *I. indigotica* at 300 mmol·L^−1^, (**d**) Fresh weight of aerial part of *I. indigotica* at 0, 25, 300 mmol·L^−1^, (**e**) Tiller number of *I. indigotica* at 0, 25, 300 mmol·L^−1^, (**f**) Plant height of *I. indigotica* at 0, 25, 300 mmol·L^−1^. Different * represent significant differences (* *p* < 0.05, ** *p* < 0.01, and *** *p* < 0.001) compared with the control group.

The effect of salt stress on the growth morphology of *I. indigotica* leaves is shown in Figure 1. At a salt concentration of 300 mmol·L^−1^, the leaves of *I. indigotica* began to become smaller, shorter, or deformed, as shown in Figure 1c. The high-salt environment caused the leaves of the aboveground part of *I. indigotica* to turn yellow or even begin to decay. Stress induced by high salt concentrations can also lead to a decrease in the fresh weight of the aerial part of *I. indigotica*. As shown in Figure 1b, low-salt-concentration stress promoted plant growth, whereas high-salt-concentration stress inhibited it. For example, when the salt concentrations were 25 and 300 mmol·L^−1^, the fresh weight of the aerial part of *I. indigotica* increased by 61.88% and decreased by 24.84%, respectively, and the differences were significant (*p* < 0.05).

Salt also has a negative effect on the development of the reproductive organs and lateral branches of *I. indigotica*, resulting in changes in the number of tillers. As shown in Figure 1e, when the salt concentration was 300 mmol·L^−1^, the tiller number of *I. indigotica* decreased by 52.63% compared with that of the control group, and the difference was significant (*p* < 0.05). Besides, salt stress led to a decrease in the plant height of *I. indigotica*, as shown in Figure 1f, because salt has a limiting effect on the overall growth and development of plants. When the salt concentrations were 25 and 300 mmol·L^−1^, the plant height of *I. indigotica* increased by 18.4% and decreased by 20.23%, respectively, compared with that of the control group, and these differences were significant (*p* < 0.05).

### 2.2. Effects of Salt Stress on Underground Part

**Figure 2:**
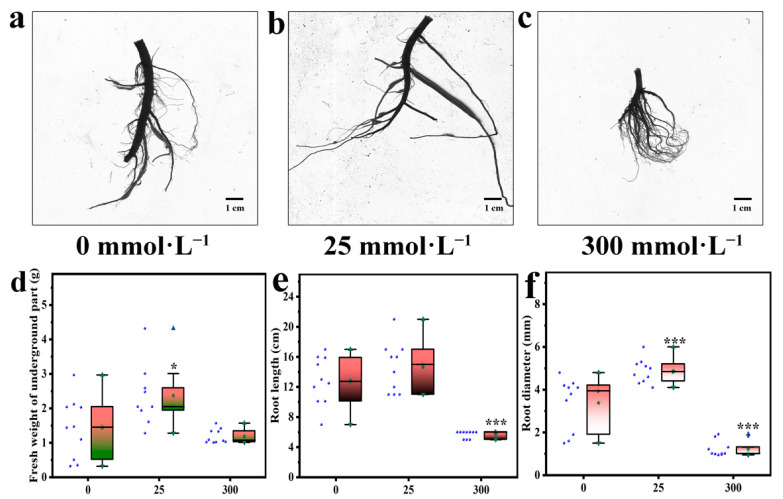
Underground part of *I. indigotica* under stress induced by different concentrations of salt. (**a**) Root morphology of *I. indigotica* at 0 mmol·L^−1^, (**b**) Root morphology of *I. indigotica* at 25 mmol·L^−1^, (**c**) Root morphology of *I. indigotica* at 300 mmol·L^−1^, (**d**) Fresh weight of underground part of *I. indigotica* at 0, 25, 300 mmol·L^−1^, (**e**) Root length of *I. indigotica* at 0, 25, 300 mmol·L^−1^, (**f**) Root diameterr of *I. indigotica* at 0, 25, 300 mmol·L^−1^. Different * represent significant differences (* *p* < 0.05, ** *p* < 0.01, and *** *p* < 0.001) com-pared with the control group.

The effect of salt stress on the root structure of *I. indigotica* is shown in Figure 2a–c. Salt stress can lead to a decrease in the fresh weight of the underground part of *I. indigotica*, mainly because the accumulation of salt in soil leads to decreased soil water potential, which, in turn, affects the water-absorption and nutrient-absorption capacities of the roots and limits the growth rate and length of the roots. Moreover, a high-salt environment can lead to an imbalance of osmotic regulation inside and outside cells, causing cell atrophy and water loss, which, in turn, affect the morphological structure of the root. This effect may lead to root fracture and deformation. The effect of salt stress on the fresh weight of the underground part of *I. indigotica* is shown in Figure 2d. When the salt concentration was 300 mmol·L^−1^, the fresh weight of the underground part of *I. indigotica* decreased by 17.14% compared with that of the control group. Salt stress can inhibit the root growth of *I. indigotica*. A high-salt environment can lead to water loss from cells, affect the metabolic activity of root cells, and limit the growth rate and length of roots. As shown in Figure 2e, salt stress caused root length to first increase and then decrease.

When the salt concentration was 300 mmo·L^−1^, root diameter decreased by 55.68% compared with that of the control group, and the difference was significant (*p* < 0.05). A salt environment can lead to water loss in root cells and disrupt the water-[potential balance in plant cells. This phenomenon causes the root cells to shrink and contract, which, in turn, causes the root diameter to become smaller. Figure 2f shows that salt stress had a great influence on the root diameter of *I. indigotica*. When the salt concentrations were 25 and 300 mmol·L^−1^, the fresh weight of the aboveground part of *I. indigotica* increased by 55.92% and decreased by 63.85%, respectively, compared with that of the control group, with significant differences (*p* < 0.05).

### 2.3. Effects of Salt Stress on Antioxidant System in Roots of I. indigotica

**Figure 3:**
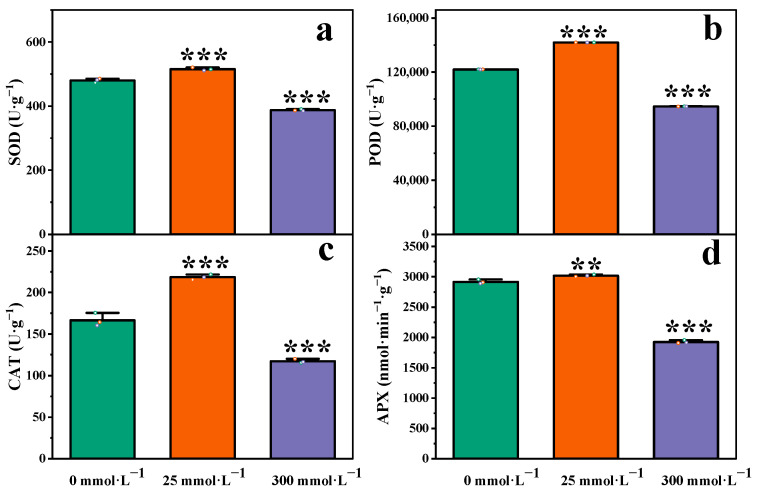
Changes in the antioxidant system of *I. indigotica* under stress induced by different concentrations of salt. (**a**) SOD activity of *I. indigotica* at 0, 25, 300 mmol·L^−1^, (**b**) POD activity of *I. indigotica* at 0, 25, 300 mmol·L^−1^, (**c**) CAT activity of *I. indigotica* at 0, 25, 300 mmol·L^−1^, (**d**) APX activity of *I. indigotica* at 0, 25, 300 mmol·L^−1^. Different * represent significant differences (* *p* < 0.05, ** *p* < 0.01, and *** *p* < 0.001) compared with the control group.

In the roots of *I. indigotica*, the activity of POD increased in soil with 25 mmol·L^−1^ NaCl and decreased gradually in soil with 300 mmol·L^−1^ NaCl, and these differences were significant (*p* < 0.05, Figure 3a). When the salt concentrations were 25 and 300 mmol·L^−1^, the levels of POD activity in the roots of *I. indigotica* were 1.16 and 0.78 times that in the control group, respectively.

Figure 3b shows the changes in superoxide SOD activity under salt stress. The SOD activity increased under stress induced by low concentrations of salt and decreased in high-salt concentrations. When the NaCl concentration was 25 mmol·L^−1^, the SOD activity in the root of *I. indigotica* increased by 7.36% compared to that of the control group. When the NaCl concentration was 300 mmol·L^−1^, the SOD activity was lower (a decrease of 19.30%) than that of the control group. At NaCl concentrations of 25 and 300 mmol·L^−1^, the SOD activity was significantly different compared with that in the control group.

The results of the test for CAT activity in the root of *I. indigotica* are shown in Figure 3c. With the stress induced by the increase in NaCl concentration, the CAT activity first increased and then decreased. When the NaCl concentration was 25 mmol·L^−1^, the CAT activity increased to 1.31 times that of the control group. When the NaCl concentration was 300 mmol·L^−1^, the CAT activity decreased to 0.70 times that of the control group.

According to the results shown in Figure 3d, the activity of APX changed compared with that in the control group to different degrees under stress induced by different concentrations of NaCl, and the difference was significant (*p* < 0.05). The APX activity increased slowly with the increase in NaCl concentration and then decreased slowly. When the NaCl concentrations were 25 and 300 mmol·L^−1^, the levels of APX activity in the roots of *I. indigotica* were 1.03 and 0.66 times that in the control group, respectively.

### 2.4. Effect of Salt Stress on Ion Content in Roots of I. indigotica

**Figure 4:**
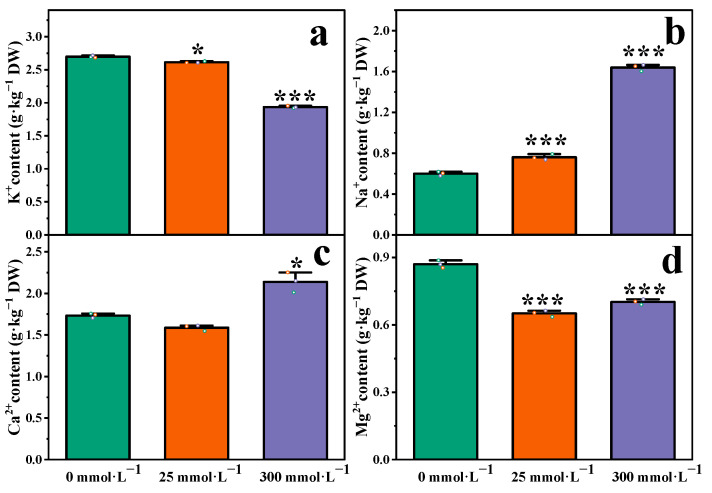
Ion contents in the roots of *I. indigotica*. (**a**) K^+^ content of *I. indigotica* at 0, 25, 300 mmol·L^−1^, (**b**) Na^+^ content of *I. indigotica* at 0, 25, 300 mmol·L^−1^, (**c**) Ca^2+^ content of *I. indigotica* at 0, 25, 300 mmol·L^−1^, (**d**) Mg^2+^ content of *I. indigotica* at 0, 25, 300 mmol·L^−1^. Different * represent significant differences (* *p* < 0.05, ** *p* < 0.01, and *** *p* < 0.001) compared with the control group.

The contents of K^+^, Na^+^, Ca^2+^, and Mg^2+^ in the roots of *I. indigotica* changed under different NaCl treatments, as shown in Figure 4. The root K^+^ content under the influence of salt stress is shown in Figure 4a. The K^+^ content was significantly lower in plants under salt stress than in the control group. When the NaCl concentrations were 0 and 25 mmol·L^−1^, the content of K+ decreased slowly. When the concentrations of NaCl were 25 and 300 mmol·L^−1^, the content of K^+^ decreased rapidly. When the NaCl concentrations were 25 and 300 mmol·L^−1^, the K^+^ content in the roots decreased by 3.10% and 28%, respectively, compared with that in the control group.

The content of Na^+^ in roots under salt stress is shown in Figure 4b, and the direction of change was opposite that of the change in K^+^ content. The Na^+^ content of *I. indigotica* roots under salt stress was significantly higher than that of the control group, and the trend was obvious. In concentrations of 0, 25, and 300 mmol·L^−1^ NaCl, the Na^+^ content continued to increase rapidly. When the NaCl concentrations were 25 and 300 mmol·L^−1^, the Na^+^ contents in the roots were respectively 1.27 and 2.87 times that of the control group. This finding indicates that salt stress could significantly increase the accumulation of Na+ in the roots of *I. indigotica*.

The changes in Ca^2+^ and Mg^2+^ are illustrated in Figure 4c,d. Under the condition of salt stress, the concentrations first decreased and then increased. When the NaCl concentration was 300 mmol·L^−1^, the difference between the two was significant. This finding suggested that the effects of Ca^2+^ and Mg^2+^ on the roots of *I. indigotica* changed significantly in high concentrations of NaCl. A notable detail that the change may be related to the regulation of ion channels in roots under salt stress. Further research could help further elucidate these differences and explore the mechanism by which salt stress affects plant-root nutrient elements.

### 2.5. Effects of Salt Stress on the Microstructure in Roots of I. indigotica

**Figure 5:**
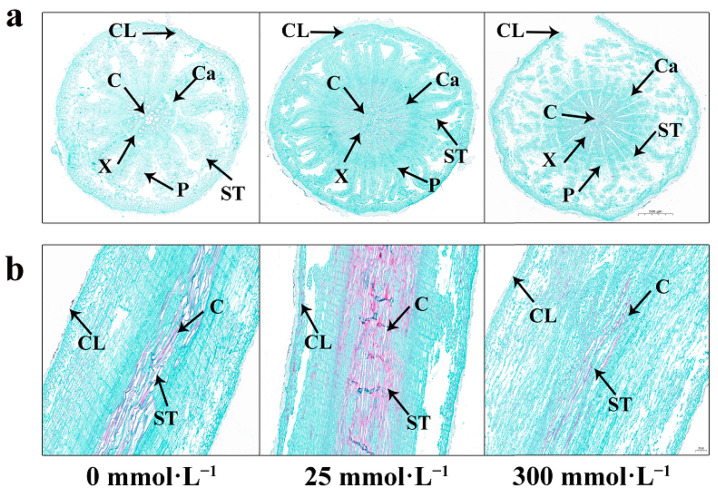
Changes in root transverse and longitudinal sections of *I. indigotica* under stress induced by different concentrations of salt. (**a**) Root transverse section; (**b**) Root longitudinal section. CL—cork layer, C—catheter, X—xylem, P—phloem, ST—sieve tube, Ca—cambium.

Salt stress had a series of effects on the root cork layer, vessel, sieve tube, xylem, phloem, and cambium of plants (Figure 5). When the NaCl concentration was 300 mmol·L^−1^, the cork layer of the roots significantly thickened compared with that of the control group to reduce the loss of water and damage to the internal tissues. The diameter of the xylem of the root decreased, and the vessels in the xylem shortened and thickened, showing an aggregated distribution, thereby improving water-transport efficiency and water-transport safety. The phloem began to thicken; the sieve tubes were broken and arranged irregularly, and the cambium stopped growing. However, these changes began to reverse when the NaCl concentration was 25 mmol·L^−1^, indicating that the lower-salt environment promoted plant growth. On the contrary, a higher-salt environment had an adverse effect on plant growth. These results indicated the effect of salt stress on root-tissue structure and the promotion or hindrance of plant growth by low- and high-salt environments. Further studies could help further elucidate the biological significance and mechanism of these changes.

### 2.6. Effects of Salt Stress on Ultrastructure of Mesophyll Cells of I. indigotica

**Figure 6:**
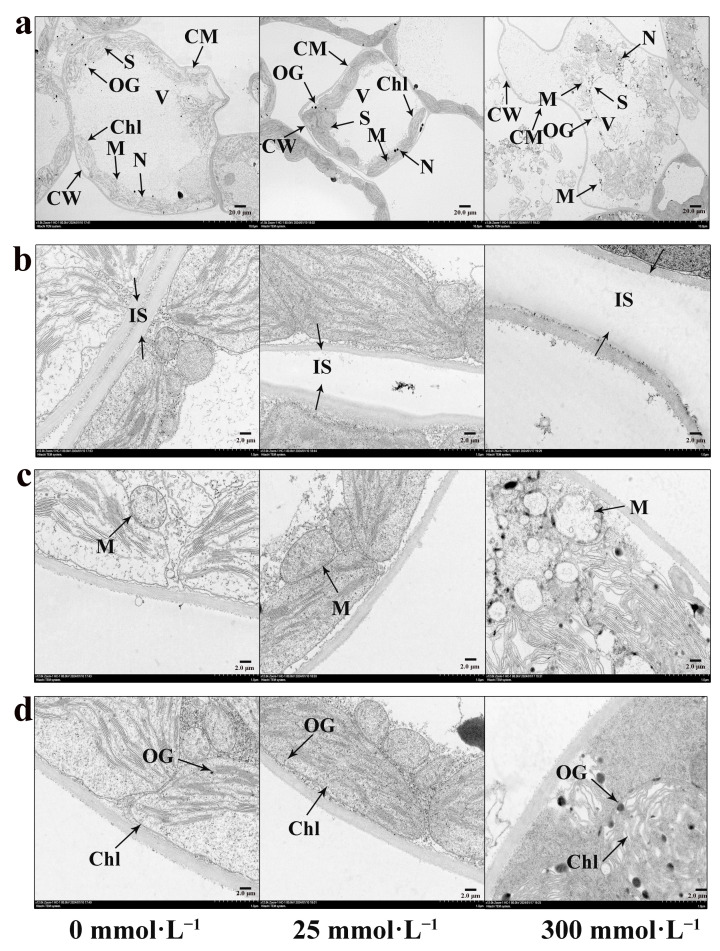
Changes in the ultrastructure of mesophyll cells of *I. indigotica* under stress induced by different concentrations of salt. (**a**) Complete mesophyll cells; (**b**) intercellular space; (**c**) mitochondrial ultrastructure; (**d**) chloroplast ultrastructure. CM—ceull membrane, OG—osmophilic granules, S—starch granule, CW—cell wall, Chl—chloroplast, V—vacuole, M—mitochondria, N—nucleus, IS—intercellular space.

In the control group (Figure 6a), the structure of the mesophyll cells of *I. indigotica* remained intact. Various organelles were neatly arranged inside the mesophyll cells. The large vacuole was located in the center of the cell; the vacuole membrane was intact; and the liquid in the vacuole was full. The chloroplasts were distributed at the edge of the cell wall and closely connected to the cell wall. In addition, the numbers of osmiophilic granules and starch granules were small. However, under stress induced by a high concentration of salt (Figure 6a), the cell wall and cell membrane began to separate and the cell wall became thin, folded, and distorted. The arrangement of various organelles became looser and began to crack. The proportion of vacuoles decreased, and the number and size of osmiophilic and starch granules increased. These changes indicated that salt stress had a significant effect on the structure and tissue of *I. indigotica* mesophyll cells. The separation and thinning of cell walls and cell membranes may lead to cell damage. Loose arrangement and lysis of organelles may affect cell function and metabolism. The changes in vacuoles, osmiophilic granules, and starch granules may be related to cells’ water regulation and water-storage metabolism. Taken together, these changes are adaptive adjustments made by plants in response to salt stress.

#### 2.6.1. Effect of Salt Stress on the Intercellular Space of *I. indigotica*

The change in the intercellular space in leaves is one of the characteristics of salt stress. Figure 6b shows that with the increase in NaCl concentration, the intercellular space of mesophyll cells gradually expanded because salt stress leads to intracellular water loss, cell-wall relaxation, and expansion. It also affects the extracellular matrix in the intercellular space, resulting in the expansion of the plant intercellular space.

#### 2.6.2. Effect of Salt Stress on the Mitochondrial Ultrastructure of *I. indigotica*

The response of mitochondria to salt stress is one of the important strategies by which plants adapt to salt environments. Before salt-stress treatment (Figure 6c), the structure of the mitochondria was nearly circular, full in shape, and complete. The mitochondria were filled, and the cristae structure was clear. The mitochondrial bilayer membrane was visible and showed a clear boundary with the cytoplasm. However, when the concentration of NaCl used in the salt-stress treatment was 25 mmol·L^−1^ (Figure 6c), the mitochondria began to become oblate, and the boundary between the overall structure and the cytoplasm became blurred. Some mitochondrial-membrane structures lost their integrity, and some even fused with the cytoplasm, resulting in the outflow of the mitochondrial internal matrix. The structure of most ridges became blurred. When the concentration of NaCl used to induce salt stress reached 300 mmol·L^−1^ (Figure 6c), the mitochondria no longer had complete membrane structures. The outflow of the matrix was further aggravated; no visible ridge structure could be seen; and most of the interior was hollow.

#### 2.6.3. Effect of Salt Stress on the Chloroplast Ultrastructure of *I. indigotica*

In the control group (Figure 6d), the mesophyll chloroplasts were fusiform, full in shape, and complete in structure. The grana lamellae were orderly and arranged in parallel. The The thylakoids are arranged neatly was neat, and the membrane-system structure of the whole chloroplast was complete. However, when the concentration of NaCl used to induce salt stress treatment was 25 mmol·L^−1^ (Figure 6d), the chloroplast began to deform and part of the membrane structure became blurred. The basal lamellae were loose, deformed, and fractured. In addition, the number of lipid droplets increased, and starch granules began to accumulate. When the concentration of NaCl used to induce salt stress treatment was 300 mmol·L^−1^ (Figure 6d), the chloroplast structure was more severely damaged. The chloroplasts further swelled and deformed, completely losing their structural integrity. The granule lamellae showed fracture and distortion. The number of osmium particles increased significantly. These results indicated that NaCl salt stress had a significant effect on the structure of mesophyll chloroplasts. With the increase in salt concentration, the morphology of the chloroplasts gradually became abnormal, and the integrity of the membrane system was destroyed. The grana lamellae became loose and fractured, and they lost their original orderly arrangement. Furthermore, the number of lipid globules and starch granules changed, which may be related to the abnormality of cell metabolism. These changes may affect photosynthesis and other functions of chloroplasts and, in turn, affect plant growth and adaptability.

### 2.7. Effects of Salt Stress on the Ultrastructure of Mesophyll Cells of I. indigotica

**Figure 7:**
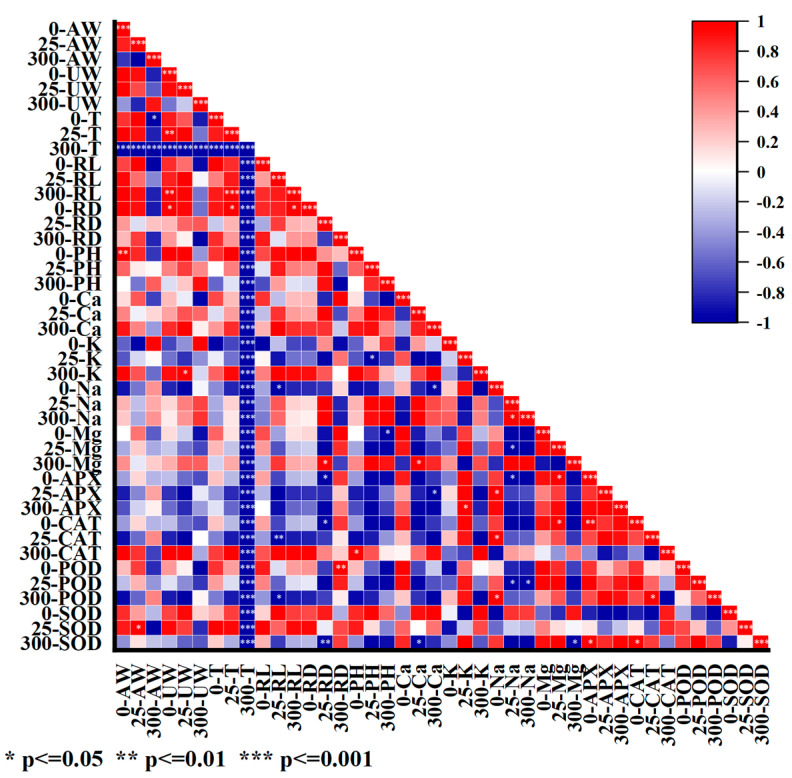
Heat map of physiological and biochemical correlations under salt stress. 0/25/300-AW: fresh weight of aerial part at 0/25/300 mmol·L^−1^ NaCl; 0/25/300-UW: fresh weight of underground part at 0/25/300 mmol·L^−1^ NaCl; 0/25/300-T: tiller number at 0/25/300 mmol·L^−1^ NaCl; 0/25/300-RL: root length at 0/25/300 mmol·L^−1^ NaCl; 0/25/300-RD: root diameter at 0/25/300 mmol·L^−1^ NaCl; 0/25/300-PH: plant height at 0/25/300 mmol·L^−1^ NaCl; 0/25/300-Ca^2+^: Ca^2+^ content at 0/25/300 mmol·L^−1^ NaCl; 0/25/300-K^+^: K^+^ content at 0/25/300 mmol·L^−1^ NaCl; 0/25/300-Na^+^: Na^+^ content at 0/25/300 mmol·L^−1^ NaCl; 0/25/300-Mg^2+^: Mg^2+^ content at 0/25/300 mmol·L^−1^ NaCl; 0/25/300-APX: APX activity at 0/25/300 mmol·L^−1^ NaCl; 0/25/300-CAT: CAT activity at 0/25/300 mmol·L^−1^ NaCl; 0/25/300-POD: POD activity at 0/25/300 mmol·L^−1^ NaCl; 0/25/300-SOD: SOD activity at 0/25/300 mmol·L^−1^ NaCl.

Figure 7 shows that stress induced by salt at a concentration of 300 mmol·L^−1^ was significantly negatively correlated with the measured physiological and biochemical parameters. Under this condition, the antioxidant enzyme activity, ion content, root length, root diameter, plant height, and plant fresh weight all decreased. A significant positive correlation was found between 25-SOD and 25-AW, and 25-POD and 25-Na were significantly negatively correlated. Moreover, 25-CAT was significantly positively correlated with 25-RL. A significant negative correlation was found between 0-Mg and 0-APX as well. Meanwhile, a significant negative correlation was found between 25-Mg and 25-Na and a significant negative correlation was observed between 0-Mg and 300-PH. Moreover, 300-Na and 25-Na were significantly negatively correlated. A significant negative correlation was found among 0-Na and 25-RL, and a significant negative correlation was found between 25-K and 25-PH. 0-PH was significantly positively correlated with 0-AW. A significant positive correlation was observed between 0-RD and 0-UW.

## 3. Discussion

The aerial parts of plants include the stems, leaves, and flowers. Each part undertakes different functions and tasks and cooperates to complete the growth and life activities of plants. The main function of stems is to support plants and transport water and nutrients. The main function of leaves is to perform photosynthesis, absorb carbon dioxide, and release oxygen. The main function of flowers is to reproduce [23,24]. Figure 1 shows that 300 mmol·L^−1^ NaCl reduced the biomass of the aerial part of *I. indigotica*, indicating that the growth of the plant was inhibited. This effect may occur because salt interferes with the synthesis of chlorophyll and the activity of the chlorophyll enzyme, resulting in the color of the leaves becoming yellowish or in the development of spots [5]. The damage to plants caused by salt stress plants mainly results from osmotic stress, ion toxicity, and nutritional imbalance, but it is ultimately manifested as plant growth inhibition, plant dwarfing, leaf yellowing, infection with serious diseases, infestation by insect pests, and even death [25,26]. Moreover, related studies have shown that salt stress can cause walnut seedling leaves to turn yellow, wither, curl, and fall off, consistent with the results of the present study [27].

Biomass and plant morphology are some of the most direct indicators for assessing the degree of salt stress and plant resistance [28]. Salt stress also leads to a decrease in the number of tillers of plants. A high-salt environment can interfere with the physiological processes of plants such as water absorption and transportation, thus affecting their growth and development. This phenomenon may lead to the normal development of the stem base and the blocking of tillering point, thereby reducing the number of tillers. With the increase in NaCl concentration, this inhibition phenomenon could become more obvious [29]. In this experiment, a low level of salt stress had a slight effect on the growth of *I. indigotica*, but when the concentration of NaCl treatment was 300 mmol·L^−1^, the fresh weight of the roots, stems, and leaves and the height and tiller number of plants decreased significantly. The plants shortened and the leaf color lightened. This finding indicates that Isatis *I. indigotica* has a degree of salt tolerance.

The problem of global soil salinization is becoming increasingly serious and has a great effect on crop yield. Plants exhibit a series of responses, including remodeling of their root structures, to cope with salt stress [30]. The results of the present study (Figure 1 and Figure 2) show that low concentrations of salt stress can increase the fresh weight of the underground part of the plant, promote root growth, and increase the diameter of the root. However, high concentrations of salt stress led to a decrease in the fresh weight of the underground part of the plant, inhibited root growth, and reduced root diameter. This finding is consistent with other research results [31,32].

Under salt stress, the metabolism of plant leaf cells can become disordered, and antioxidant enzymes play a role in scavenging harmful free radicals and oxidizing substances in cells [33,34]. SOD can convert superoxide radicals (O2·^−^) into more stable H_2_O_2_ molecules to prevent the damage superoxide radicals can cause to intracellular structures and molecules [35]. CAT can decompose intracellular H_2_O_2_ into H_2_O and O_2_, thereby reducing the toxicity of H_2_O_2_ to cells [36]. POD can reduce the accumulation of harmful oxidizing substances and maintain the redox balance in cells, thus protecting biological molecules [37]. APX can neutralize H_2_O_2_, protect cells from free radicals and oxidizing substances, and maintain the normal function of cells and organisms [38]. In the present study, the activities of SOD, POD, CAT, and APX showed a trend of increasing first and then decreasing at 0, 25, and 300 mmol·L^−1^ NaCl (Figure 3). A similar trend was confirmed in a study of seedlings of *P. euphratica* × *P. variegata* [39], indicating that the number of free radicals produced by high-concentration salt stress exceeded the scavenging capacity of antioxidant enzymes and that the damage caused by salt stress to plants thus increased. In addition, comparison of the four antioxidant enzymes showed that the POD had the highest activity, which indicated that *I. indigotica* enhanced tolerance by increasing the POD activity in a salt-stress environment. These changes may be related to the gene-expression levels of plants, and further research is needed.

Under the influence of salt damage, excessive intake of sodium ions (Na^+^) by plants inhibits the absorption and distribution of potassium (K^+^), calcium (Ca^2+^), and magnesium ions (Mg^2+^) and destroys the homeostasis of ions [40]. In plants, K^+^ is the main cellular cation and is an essential nutrient [41]. The present study found that under salt-stress conditions, the content of K^+^ showed a continuous downward trend, the content of Na^+^ showed a continuous upward trend, and the content of Ca^2+^ and Mg^2+^ decreased first and then increased (Figure 4). This finding is consistent with the results of a study on the saline–alkali tolerance of willow [42].

The structure and function of plant cells are closely related. These structures cooperate with one another to form a highly coordinated and complementary system that enables cells to complete various physiological functions. The relationship between cell structure and function ensures that plant cells can carry out life activities such as photosynthesis, material transport, growth and development, and adaptation to the environment [43]. Therefore, understanding the microscopic changes that occur in roots and the ultrastructural changes that occur in mesophyll cells after salt stress is important. In the present study, with the increase in NaCl concentration, the thickness of the root cork layer of *I. indigotica* increased, the diameter of the xylem decreased, the vessel shortened and thickened, the phloem thickened, the sieve tube ruptured, and the cambium stopped growing (Figure 5). Other studies have reached similar conclusions, and the results of a study on the changes in plant microstructure under salt stress showed agreement [44].

According to the results related to leaf ultrastructure, with the increase in NaCl treatment concentration, the organelles in the mesophyll became distorted or even disintegrated. The numbers of starch granules and osmiophilic granules increased significantly (Figure 6) to effectively adsorb salt ions, scavenge reactive oxygen species, and promote plant growth [45]. Similar changes have also been observed in other plants [46]. The structures of chloroplasts and mitochondria were seriously damaged. The mitochondria flattened; the membrane structure was incomplete; the matrix outflow increased; and the ridge structure became blurred or even disappeared. These changes may affect mitochondrial function, including energy production and cell metabolism. Considering that the mitochondria are the energy centers in cells, the ridge structure on the inner membrane provides a larger surface area, which is conducive to energy generation by cells. Therefore, changes in mitochondrial structure may reduce the capacity of plants for energy production and hurt the normal function of cells. In general, these mitochondrial changes reveal the adaptation mechanism used by plants in the face of salt stress. The chloroplast of a leaf cell is mostly kidney-shaped, spindle-shaped, or arched-shaped. Under salt stress, the shape of the chloroplast becomes irregular or nearly circular. The internal structure of the chloroplast thylakoids changed; the number began to decrease; the volume expanded; and the internal protein structure changed. These changes are related to a mechanism that allows *I. indigotica* to adapt to a high-salt environment during its growth. Similar ultrastructural changes have been found in other plants such as Robinia pseudoacacia [47], wheat [48], and Quercus dentata [49]. In addition, under high degrees of salt stress, the cell structure of these plants has also undergone serious changes [50,51]. In a low-salt environment, the effect on the mitochondria and chloroplasts is relatively light, whereas in a high-salt environment, the effect is serious.

## 4. Materials and Methods

### 4.1. Effect of Salt Stress on Ion Content

*I. indigotica* seeds were purchased from the Hebei Anguo market. *I. indigotica* seeds of same size were evenly sown in nutrient soil for a total of 15 pots with 50 seeds per pot. After 1 week of germination, NaCl (0, 25, and 300 mmol·L^−1^) treatment was started, with five replicates per treatment. The seeds were regularly watered every day at 9:00 a.m. with 2 L of water per pot, and the Hoagland nutrient solution was used to configure salt water. The stepwise watering method was used to increase the salt concentration by 25 mmol·L^−1^ every day until the final concentration was reached to avoid a salt-shock reaction. After the final salt concentration was reached, the plants continued to grow for 4 weeks and then were sampled.

### 4.2. Measurement of Aerial and Underground Part Growth Indices

After 4 weeks of growth, the intact plants were taken from the soil, washed with distilled water to remove the sediment on the surface, and then wiped dry with filter paper. The aboveground and underground parts of the plant were weighed separately, and the number of tillers was recorded. The plant height was measured, and the root diameter and root length were measured using a vernier caliper. Each index was measured 10 times.

### 4.3. Determination of the Activities of Antioxidant Enzymes

The activities of SOD, POD, CAT, and APX in the roots of *I. indigotica* were detected using a kit (Nanjing Jiancheng Biological Research Institute, Nanjing, China).

SOD activity: At a ratio of plant-tissue mass (g):volume (mL) = 1:9, nine times the volume of normal saline was added to the plant tissue and 10% tissue homogenate was prepared. Following the steps indicated on the kit, we sequentially added 1.0 mL of reagent I, 0.05 mL of tissue homogenate, 0.1 mL of reagent II, 0.1 mL of reagent III, and 0.1 mL of reagent Ⅳ. This solution was allowed to react for 40 min at 37 °C in a water bath, and 2 mL of the developer was added. The solution was stored at room temperature for 10 min, and OD_550_ was used to measure absorbance with distilled water as a control. The results were calculated according to fresh weight.

POD activity: At a ratio of plant-tissue mass (g):volume (mL) = 1:9, nine times the volume of normal saline was added to the tissue and 10% tissue homogenate was prepared in an ice-water bath. Next, the plant-tissue homogenate was centrifuged at 3000 rpm for 10 min. Following the steps indicated on the kit, we sequentially added 2.4 mL of reagent I, 0.3 mL of reagent II, 0.2 mL of reagent III, and 0.1 mL of extract to the reaction system. The system was allowed to react for 30 min at 37 °C in a water bath. Next, 1.0 mL of reagent IV was added, and the sample was mixed well and centrifuged for 10 min at 3500 rpm. The OD_420_ of the supernatant was used to measure absorbance with distilled water as a control. The results were calculated according to fresh weight.

CAT activity: At a ratio of plant-tissue mass (g):volume (mL) = 1:9, nine times the volume of normal saline was added to the tissue, and 10% tissue homogenate was prepared under ice water bath conditions, the plant-tissue homogenate was centrifuged at 2500 rpm for 10 min. Following the steps indicated on the kit, we sequentially added 0.05 mL of extract to the reaction system, 1.0 mL of reagent I (preheating at 37 °C) and 1.0 mL of reagent II (preheating at 37 °C). The solution was allowed to react for 1 min at 37 °C. Next, 1.0 mL of reagent III and 0.1 mL of reagent IV were added, and OD_405_ was used to measure absorbance with distilled water as a control. The results were calculated according to fresh weight.

APX activity: At a ratio of plant-tissue mass (g):volume (mL) = 1:9, nine times the volume of normal saline was added to the tissue and 10% tissue homogenate was prepared in an ice-water bath. The plant tissue was centrifuged at 10,000 rpm for 10 min. Following the steps indicated on the kit, we sequentially added 100 μL of extract, 700 μL of reagent I, 100 μL of reagent II, and 100 μL of reagent III to the reaction system. The supernatant was removed and tested. OD_290_ was used to measure absorbance with distilled water as a control. The results were calculated according to fresh weight.

### 4.4. Extraction and Quantification of Ion Contents

The root tissue of *I. indigotica* was washed with sediment and dried with a filter paper at 60 °C until a constant weight was reached. The dried sample was ground into powder, and 0.3 g of the sample was weighed and added to 30 mL of deionized water. After being shaken well, the sample was heated in a boiling-water bath for 3 h. After cooling, it was filtered and diluted to 50 mL. Reference method [51] recommended the use of atomic absorption spectrometry (Shimadzu AA-6300, Kyoto, Japan) for the determination of Na^+^, K^+^, Ca^2+^, and Mg^2+^.

### 4.5. Extraction and Quantification of Ion Contents

Fresh root tips were fixed in formalin fixative, embedded in tissue, sliced, and dewaxed in water. After dewaxing, the sections were stained in safranine dye solution for 3–5 s, washed with tap water to remove excess dyes, and then decolored in 50%, 70%, and 80% gradient alcohol for 3–8 s individually. Afterward, they were stained in fast green dye solution for 4–6 s, dehydrated in three cylinders of absolute ethanol, and then rendered transparent by immersion in clean xylene for 5 min. A neutral gum was used to seal the film, and an optical microscope (Nikon Eclipse E100, Kyoto, Japan) was used for imaging (Nikon DS-U3, Kyoto, Japan).

A sample of fresh tissue was placed in the electron-microscope fixative (Servicebio-G1102, Wuhan, China) and cut into 1 mm^3^ pieces with a scalpel. The cut tissue was fixed with 1% osmic acid at room temperature for 7 h in the dark. After fixation, the tissue was dehydrated at room temperature and infiltrated. After embedding, the samples were polymerized in an oven at 60 °C for 48 h. After the polymerization was completed, the samples were cut into ultrathin slices of 60–80 nm by an ultrathin slicing machine, and the slices were fished out using a 150-mesh square film copper mesh. The copper mesh and sections were placed in a 2% uranium acetate-saturated alcohol solution and stained in the dark for 8 min. Subsequently, 70% alcohol was used to clean the samples three times; then, ultrapure water was used to clean the samples three times. The sections were stained in 2.6% lead citrate solution for 8 min, washed with ultrapure water three times, and finally gently dried with filter paper. The copper mesh slices were placed into a copper mesh box and dried at room temperature overnight. The sections were observed using a transmission electron microscope (Hitachi HT7800, Kyoto, Japan), and images were collected for further analysis.

### 4.6. Statistical Analysis

SPSS (version 26.0) software was used for statistical analysis of relevant data. ANOVA, followed by Duncan’s test, was performed to determine differences between groups. The level of significance was set at *p* < 0.05, and graphs were drawn using OriginPro (version 2021).

## Data Availability

The data presented in this study are available on request from the corresponding author.
